# Biomimetic Hybrid Nanofiber Sheets Composed of RGD Peptide-Decorated PLGA as Cell-Adhesive Substrates

**DOI:** 10.3390/jfb6020367

**Published:** 2015-05-29

**Authors:** Yong Cheol Shin, Jong Ho Lee, Min Jeong Kim, Ji Hoon Park, Sung Eun Kim, Jin Su Kim, Jin-Woo Oh, Dong-Wook Han

**Affiliations:** 1 Department of Optics and Mechatronics Engineering, BK21+ Nano-Integrated Cogno-Mechatronics Engineering, College of Nanoscience & Nanotechnology, Pusan National University, Busandaehak-ro 63beon-gil, Geumjeong-gu, Busan 609-735, Korea; E-Mails: choel15@naver.com (Y.C.S.); pignunssob@naver.com (J.H.L.); kdh6253@naver.com (M.J.K.); smilemippy@gmail.com (J.H.P.); 01048470363@naver.com (S.E.K.); wlstn5756@naver.com (J.S.K.); 2 Department of Nanoenergy Engineering, College of Nanoscience & Nanotechnology, Pusan National University, Busandaehak-ro 63beon-gil, Geumjeong-gu, Busan 609-735, Korea; E-Mail: ojw@pusan.ac.kr

**Keywords:** tissue engineering scaffolds, cell-adhesive substrates, hybrid nanofiber sheets, electrospinning, poly(lactic-*co*-glycolic acid), RGD peptides

## Abstract

In biomedical applications, there is a need for tissue engineering scaffolds to promote and control cellular behaviors, including adhesion, proliferation and differentiation. In particular, the initial adhesion of cells has a great influence on those cellular behaviors. In this study, we concentrate on developing cell-adhesive substrates applicable for tissue engineering scaffolds. The hybrid nanofiber sheets were prepared by electrospinning poly(lactic-*co*-glycolic acid) (PLGA) and M13 phage, which was genetically modified to enhance cell adhesion thru expressing RGD peptides on their surface. The RGD peptide is a specific motif of extracellular matrix (ECM) for integrin receptors of cells. RGD peptide-decorated PLGA (RGD-PLGA) nanofiber sheets were characterized by scanning electron microscopy, immunofluorescence staining, contact angle measurement and differential scanning calorimetry. In addition, the initial adhesion and proliferation of four different types of mammalian cells were determined in order to evaluate the potential of RGD-PLGA nanofiber sheets as cell-adhesive substrates. Our results showed that the hybrid nanofiber sheets have a three-dimensional porous structure comparable to the native ECM. Furthermore, the initial adhesion and proliferation of cells were significantly enhanced on RGD-PLGA sheets. These results suggest that biomimetic RGD-PLGA nanofiber sheets can be promising cell-adhesive substrates for application as tissue engineering scaffolds.

## 1. Introduction

Over the past few years, tissue engineering has emerged as a promising novel approach to regenerate and substitute sophisticated tissues and organs. Recently, there have been significant efforts to fabricate tissue engineering scaffolds that can support cellular behaviors, including adhesion and proliferation. However, it is still a challenge to fabricate tissue engineering scaffolds that can support and promote cellular behaviors. Ideally, the tissue engineering scaffolds should provide an appropriate three-dimensional microenvironment for cell growth and encourage cell adhesion and proliferation. In addition, the scaffolds should also be structurally similar to the natural extracellular matrix (ECM) and be able to act as the natural ECM. Therefore, much research has been carried out to develop tissue engineering scaffolds that mimic the natural ECM by using various materials and methods.

There have been several techniques for fabricating tissue engineering scaffolds, including electrospinning, phase separation, specific template methods and self-assembly [1,2,3,4,5,6]. Among various types of scaffolds, electrospun nanofiber sheets are a promising approach for tissue engineering, because they have not only a three-dimensional porous architecture with interconnected pores, but also a very high surface-to-volume ratio. Therefore, many studies have reported that electrospun nanofiber sheets composed of many diverse biomaterials are suitable for application as tissue engineering scaffolds [6,7,8,9].

Poly(lactic-*co*-glycolic acid) (PLGA) is the most extensively-used biocompatible polymer to fabricate electrospun nanofiber sheets, because of its suitable physicochemical properties for electrospinning and good solubility in organic solvents [10,11]. In addition, PLGA has been widely investigated for tissue engineering scaffolds due to its favorable degradation behavior, well-defined characteristics in the body and approval by the U.S. Food and Drug Administration [12,13,14]. On the other hand, PLGA nanofiber sheets decorated with various biomaterials can effectively enhance cellular behaviors. RGD peptide, a tripeptide (Arg-Gly-Asp) found within ECM proteins, is one of the most attractive candidates. RGD peptide is a typical recognition motif for integrin and can promote cell adhesion, proliferation and differentiation [15]. Consequently, RGD peptide-decorated substrates have been developed as tissue engineering scaffolds [16,17].

Herein, we fabricated biomimetic hybrid nanofiber sheets composed of RGD peptide-decorated PLGA (RGD-PLGA) nanofibers via an electrospinning technique. The M13 phage is a filamentous virus that has a nanofiber-like shape (~6.6 nm in diameter and ~880 nm in length) and does not influence mammalian and human cells [18,19,20]. It is comprised of a circular single-stranded DNA covered by 2700 major coat proteins (pVIII) and can display desired proteins on its surface by genetic modification [21]. Specific peptides, displayed on the major coat proteins, are densely packed on the surface of the M13 phage, which, in turn, leads to effective production of the desired peptides. Hence, bioengineered M13 phages are widely used for bio-medical applications, such as bone tissue engineering, photodynamic therapy and gene delivery [22,23,24]. In particular, it has been reported that the bioengineered M13 phage films can be used as a scaffold for controlling and supporting the proliferation and differentiation of mesenchymal stem cells [25]. Additionally, it has been also demonstrated that the RGD peptide displaying M13 phage (RGD-M13 phage) can be employed for promoting and stimulating cellular behaviors, including adhesion, proliferation and differentiation [19,26,27]. Therefore, in the present study, the M13 phages were genetically modified to display RGD peptides on their surfaces, and the RGD-M13 phages were used to decorate the RGD peptide on the biomimetic hybrid nanofiber sheets. The RGD-PLGA nanofiber sheets were characterized by scanning electron microscopy (SEM), immunofluorescence staining, contact angle measurement and differential scanning calorimetry (DSC). Furthermore, we evaluated the cellular behaviors of four different types of cells on the RGD-PLGA nanofiber sheets to explore their possibility as cell-adhesive substrates for application as tissue engineering scaffolds.

## 2. Results and Discussion

### 2.1. Characterizations of RGD-PLGA Nanofiber Sheets

**Figure 1:**
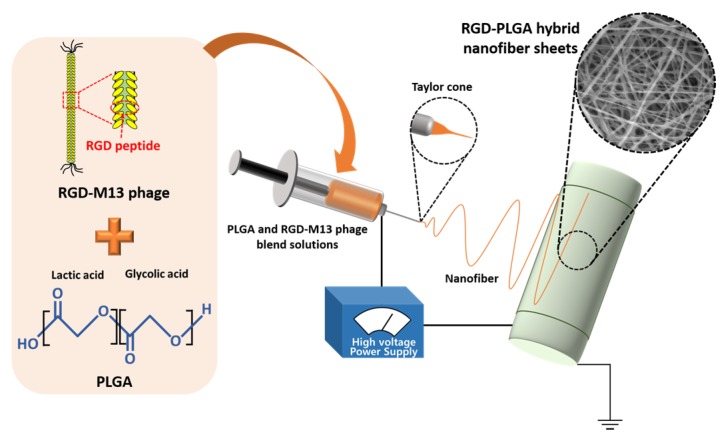
Schematic diagram of the electrospinning system for the fabrication of the RGD peptide-decorated poly(lactic-*co*-glycolic acid) (RGD-PGLA) hybrid nanofiber sheets.

**Figure 2:**
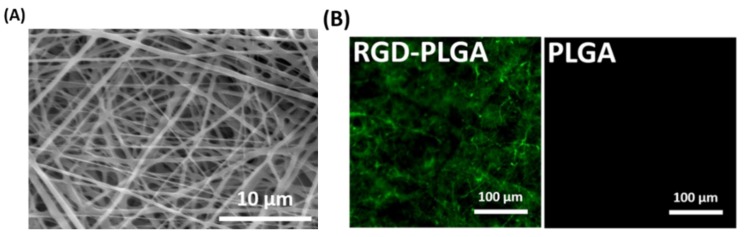
(**A**) Representative SEM image of RGD-PLGA nanofiber sheets; (**B**) representative immunofluorescence images of the pure PLGA and RGD-PLGA nanofiber sheets. RGD-M13 phages in the RGD-PLGA nanofiber sheets were immunostained with the fluorescein isothiocyanate (FITC)-labelled anti-M13 phage antibody (green). All images shown in this figure are representative of six independent experiments with similar results.

RGD-PLGA nanofiber sheets were fabricated by electrospinning of PLGA and RGD-M13 phage blend solutions (Figure 1). The optimal mixing ratio between RGD-M13 phage and PLGA solution was 1:3. The surface morphology of RGD-PLGA nanofiber sheets was observed by SEM, as shown in Figure 2A. The SEM image demonstrated that the RGD-PLGA nanofiber sheet has a three-dimensional porous architecture with interconnected pores homologous to the ECM. In addition, the RGD-PLGA nanofibers have continuous, smooth and beadless morphologies with an average diameter of 370 ± 190 nm. This indicates that the electrospinning parameters, including the mixing ratio, voltage, distance between the tip of the needle and the collector and the flow rate of a blend solution were optimum for the fabrication of RGD-PLGA nanofibers. These nanometer-scale diameters of RGD-PLGA hybrid fibers allow achieving a high surface area-to-volume ratio [28]. Therefore, the RGD-PLGA nanofiber sheets can effectively interact with cells. The distribution of decorated RGD peptides on the nanofiber sheets was examined by immunofluorescence staining for RGD-M13 phages (Figure 2B). The RGD-PLGA nanofiber sheet showed green fluorescence of RGD-M13 phages throughout the sheets, whereas the pure PLGA nanofiber sheet did not show any detectable fluorescence. Therefore, it was revealed that the RGD-PLGA nanofiber sheets were successfully prepared, and the RGD peptides were abundantly decorated on the sheets.

**Figure 3:**
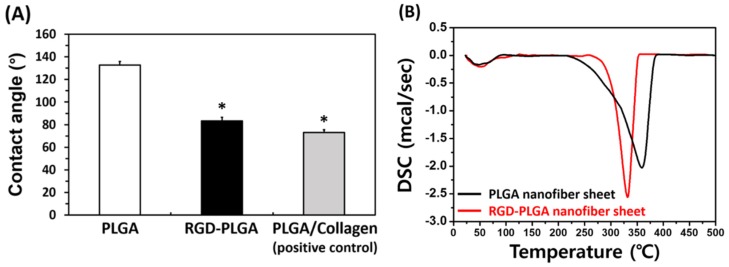
(**A**) Water contact angles of the pure PLGA, RGD-PLGA and PLGA/collagen nanofiber sheets. An asterisk (*) denotes a significant difference between the PLGA nanofiber sheet and other groups, *p* < 0.05. (**B**) DSC thermogram of the pure PLGA and RGD-PLGA nanofiber sheets.

Figure 3A shows the water contact angles of the pure PLGA, RGD-PLGA and PLGA/collagen nanofiber sheets. The water contact angles were 132.7° ± 3.2°, 83.2° ± 3.3° and 73.2° ± 2.3° for the pure PLGA, RGD-PLGA and PLGA/collagen nanofiber sheets, respectively. The water contact angles of electrospun nanofiber sheets were significantly (*p* < 0.05) decreased by blending with RGD-M13 phage or collagen. The improvement in the hydrophilicity of the RGD-PLGA nanofiber sheets is comparable to that of the PLGA/Collagen nanofiber sheets as the positive control. The increased hydrophilic nature of the substrate surface can lead to enhanced cellular behaviors, including cell adhesion, migration, proliferation and differentiation [29]. However, although collagen is a very hydrophilic biomaterial, one of the main disadvantages of collagen-based sheets is its rapid degradation behavior [30,31]. Therefore, RGD-PLGA nanofiber sheets are desirable candidates as cell-adhesive substrates, because they have a suitable architecture and a sufficient hydrophilic surface for cell adhesion. We also evaluated the thermal behaviors of PLGA and RGD-PLGA nanofiber sheets by DSC (Figure 3B). The DSC thermogram showed two endotherm peaks for each nanofiber sheets. The first endotherm peak was observed at approximately 48.1 and 50.1 °C for the pure PLGA and RGD-PLGA nanofiber sheets, respectively. This can be due to the glass transition temperature of PLGA ranging between approximately 45–55 °C [32,33]. The second endotherm peak, the endothermic melting peak, was observed at approximately 359.4 and 332.1 °C for the pure PLGA and RGD-PLGA nanofiber sheets, respectively. These results indicated that the thermal behavior of the RGD-PLGA nanofiber sheets is not substantially affected by RGD-M13 phage addition under cell culture conditions, although their thermal stability was slightly decreased at temperatures higher than 300 °C. Therefore, it is suggested that the RGD-PLGA nanofiber sheets can serve as a favorable microenvironment for cell adhesion and proliferation.

### 2.2. The Initial Cell Adhesion on RGD-PLGA Nanofiber Sheets

**Figure 4:**
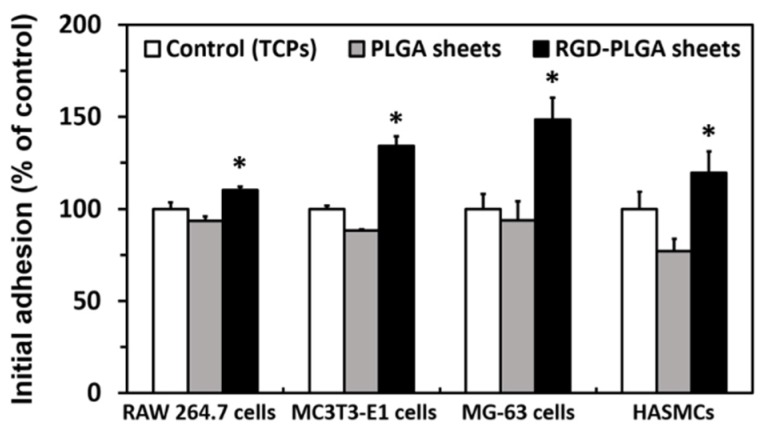
Initial adhesion of RAW 264.7 cells, MC3T3-E1 cells, MG-63 cells and primary human aortic smooth muscle cell (HASMC) on the controls (tissue culture plastics, TCPs), PLGA nanofiber sheets and RGD-PLGA nanofiber sheets. An asterisk (*) denotes a significant difference between the controls and RGD-PLGA nanofiber sheets, *p* < 0.05.

To evaluate the initial cell adhesion on RGD-PLGA nanofiber sheets, the four types of cells were cultured on the nanofiber sheets. The cells used in the present study were the murine macrophage cell line (RAW 264.7 cell), the murine preosteoblastic cell line (MC3T3-E1 cell), the human osteosarcoma cell line (MG-63 cell) and the primary human aortic smooth muscle cell (HASMC). All cells were seeded on the nanofiber sheets, and their initial adhesions were evaluated by measuring the cell viability at six hours based on the mitochondrial activity. As shown in Figure 4, all cells, regardless of cell types or species, showed the highest initial adhesion on the RGD-PLGA nanofiber sheets, whereas they showed the lowest initial adhesion on the pure PLGA nanofiber sheets. These results can be understood by considering the correlation between the cellular behaviors and the hydrophilic property of the substrate surface [29]. In addition, the decorated RGD peptides also contributed to an increase in the initial adhesion. Previous studies have shown that the RGD peptides are proven to enhance cell adhesion [34,35]. Consequently, the RGD-PLGA nanofiber sheets can enhance cell adhesion due to the improvement in the surface hydrophilicity and the decorated RGD peptides.

### 2.3. The Cell Proliferation on RGD-PLGA Nanofiber Sheets

**Figure 5:**
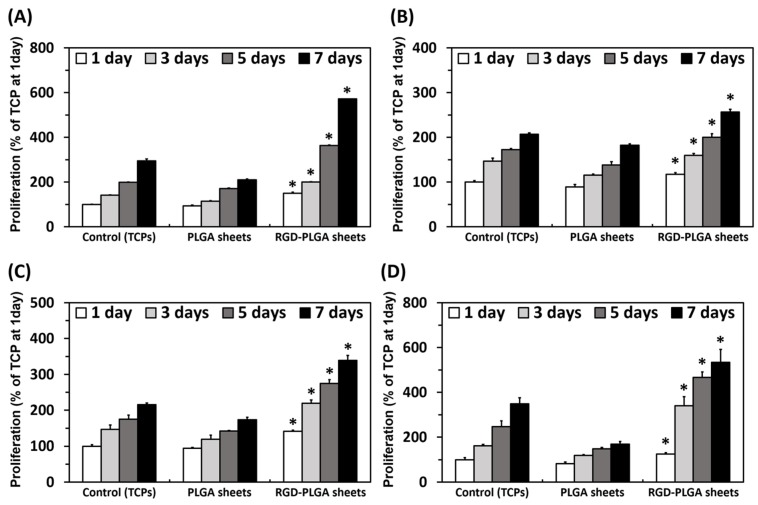
Proliferation of RAW 264.7 cells, MC3T3-E1 cells, MG-63 cells and HASMCs on the controls (TCPs), PLGA nanofiber sheets and RGD-PLGA nanofiber sheets. An asterisk (*) denotes a significant difference between the controls and RGD-PLGA nanofiber sheets, *p* < 0.05. (**A**) RAW 264.7 cells; (**B**) MC3T3-E1 cells; (**C**) MG-63 cells; (**D**) HASMCs.

We examined the proliferation of the four types of cells on the RGD-PLGA nanofiber sheets on 1, 3, 5 and 7 days after culture. The proliferation of all cells was increased with culture time (Figure 5). However, the proliferation of all cells was significantly (*p* < 0.05) increased on the RGD-PLGA nanofiber sheets by comparison with that on the pure PLGA nanofiber sheets and controls (tissue culture plastics, TCPs). These results correspond closely with previous findings showing that cell growth is effectively promoted on the RGD peptide-decorated substrates [16,17,21,36]. In addition, according to the previous literature, the promoted proliferation of cells on the RGD-PLGA nanofiber sheets could be attributed to an increase in the initial adhesion on the sheets [17]. Thus, it was found that the RGD-PLGA nanofiber sheets are highly effective at promoting the proliferation and growth of cells.

### 2.4. The Morphological Observation of HASMCs on RGD-PLGA Nanofiber Sheets

**Figure 6:**
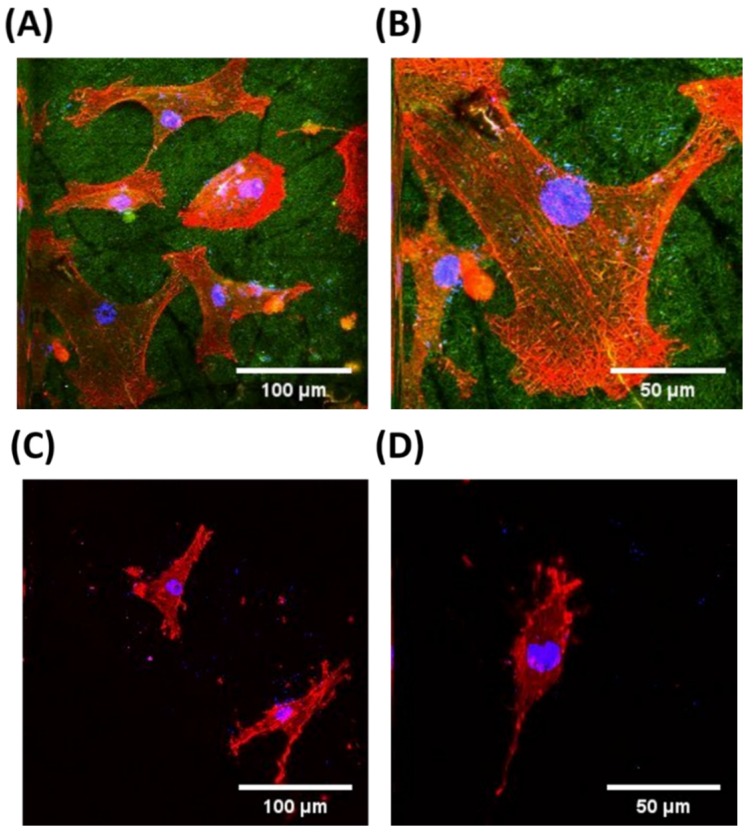
Two-photon excitation fluorescence images of HASMCs on the pure PLGA and RGD-PLGA nanofiber sheets. F-actins of HASMC cytoskeletons were stained with tetramethylrhodamine isothiocyanate (TRITC)-labelled phalloidin (red); cell nuclei were counter stained with 4',6-diamidino-2-phenylindole (DAPI, blue); and RGD-M13 phages in the RGD-PLGA nanofiber sheets were immunostained with the FITC-labelled anti-M13 phage antibody (green). All images shown in this figure are representative of six independent experiments with similar results. (**A**) Low-magnification and (**B**) higher-magnification images of HASMCs on the RGD-PLGA nanofiber sheets; (**C**) low-magnification and (**D**) higher-magnification images of HASMCs on the pure PLGA nanofiber sheets.

To confirm our findings of the promoted initial cell adhesions and proliferations, the adherent morphologies of HASMCs, cultured on the pure PLGA and RGD-PLGA nanofiber sheets for three days, were imaged with the two-photon excitation microscope. Figure 6 shows the obvious difference in HASMC morphologies between the pure PLGA nanofiber sheets and the RGD-PLGA nanofiber sheets. The HASMCs on the RGD-PLGA nanofiber sheets were successfully grown and maintained their well-adhered morphology (Figure 6A). As shown in the higher magnification image, they showed a well-organized F-actin network (Figure 6B). In addition, the RGD-PLGA nanofiber sheets exhibited green fluorescence throughout the sheet due to the decorated RGD-M13 phage. Contrary to this, the HASMCs on the pure PLGA nanofiber sheets were not fully grown and showed an irregular morphology with poorly-developed F-actins owing to the hydrophobic surface property of the sheets (Figure 6C,D). Therefore, it is suggested that the RGD-PLGA nanofiber sheets can specifically facilitate cellular behaviors, including cell adhesion and proliferation, due to the synergistic effects of an increase in the hydrophilic surface property and the RGD peptides.

## 3. Experimental Section

### 3.1. Fabrication of RGD-PLGA Nanofiber Sheets

The RGD peptides were displayed on the major coat protein of M13 phages by an inverse PCR cloning method according to the procedure described by Jin-Woo Oh and coworkers [37]. The RGD-PLGA nanofiber sheets were fabricated by using an electrospinning technique. In brief, PLGA resins (PLA/PGA = 75/25, MW = 70–110 kDa, Evonik Industries, Essen, Germany) were dissolved in 1,1,1,3,3,3-hexafluoroisopropanol (HFIP, Sigma-Aldrich Co., St Louis, MO). The RGD-M13 phage suspensions in Tris-buffered saline buffer (50 mM Tris and 150 mM NaCl, pH 7.4, Bioworld, Dublin, OH, USA) were blended with the PLGA solution. Four different compositions of RGD-M13 phage and PLGA blend solution (1:1, 1:2, 1:3 and 1:4) were prepared to determine the optimal mixing ratio of the electrospinning solution. The final concentrations of the PLGA and RGD-M13 phage were 200 and 10 mg/ml, respectively. The PLGA and RGD-M13 phage blend solution was loaded into a syringe fitted to a 25-G needle. A voltage of 14 kV was applied, and the distance between the tip of needle and the collector was 11 cm. The flow rate of the blend solution was fixed at 0.2 mL/h. Randomly-oriented RGD-PLGA hybrid nanofibers were collected on a steel rotating wheel covered with aluminum foil. After that, the RGD-PLGA nanofiber sheets were dried overnight under vacuum at room temperature (RT) in order to remove any residual solvent.

### 3.2. Characterizations of RGD-PLGA Nanofiber Sheets

The RGD-PLGA nanofiber sheets were observed under an SEM (S-800, Hitach, Tokyo, Japan). The sheets were coated with an ultrathin layer of gold/platinum by an ion sputterer (E1010, Hitach, Tokyo, Japan) prior to SEM observations. To examine the distribution of decorated RGD peptides on the nanofiber sheets, the RGD-M13 phages were immunostained with the primary anti-M13 phage antibody (Sigma-Aldrich Co., St Louis, MO, USA) and the secondary FITC-conjugated goat anti-rabbit IgG (Abcam Inc., Cambridge, MA, USA). The RGD-PLGA nanofiber sheets were incubated with the primary anti-M13 phage antibody (at 1:250 in 2% bovine serum albumin (BSA, GenDEPOT, Barker, TX, USA) solution in Dulbecco’s phosphate-buffered saline (DPBS, Gibco BRL, Rockville, MD, USA) for 2 hours at RT and then incubated with the secondary FITC-conjugated goat anti-rabbit IgG (at 1:500 in 2% BSA solution in PBS) for 1 hour at RT. The stained sheets were imaged with an Olympus IX81 inverted fluorescence microscope (Olympus Corp., Osaka, Japan). To examine the hydrophilicity of the sheets, the water contact angles of the sheets were measured by the sessile drop method using a contact angle measurement system (OCA10, Dataphysics, Filderstadt, Germany). A 10-μL sessile drop of distilled water was formed on all samples. The thermal behaviors of the nanofiber sheets were measured by DSC (MAC science, Tokyo, Japan). The nanofiber sheets were heated at a rate of 10 °C/min from 25 to 500 °C. The weight of matrices was 5.18 mg for the pure PLGA nanofiber sheets and 5.16 mg for the RGD-PLGA nanofiber sheets. Alpha-Al_2_O_3_ was used as a reference.

### 3.3. The Initial Cell Adhesion and Cell Proliferation Assays

RAW 264.7 cells, MC3T3-E1 cells, MG-63 cells and HASMCs were purchased from the American Type Culture Collection (Rockville, MD, USA) and routinely cultured in Dulbecco’s Modified Eagle’s Medium (DMEM, Welgene, Daegu, Korea), alpha-minimum essential medium (α-MEM, Welgene, Daegu, Korea), MEM with Earle’s salts (Welgene, Daegu, Korea) and smooth muscle cell growth medium-2 (SMCGM, PromoCell, Heidelberg, Germany), respectively. All cells were cultured in complete media at 37 °C in a humidified atmosphere containing 5% CO_2_ (DMEM, α-MEM and MEM were supplemented with 10% fetal bovine serum (Welgene, Daegu, Korea) and 1% antibiotic-antimycotic solution (including 10,000 units penicillin, 10 mg streptomycin and 25 μg amphotericin B per mL, Sigma-Aldrich Co., St Louis, MO, USA), and SMCGM was supplemented with 10% fetal calf serum, 0.5 ng/mL human epidermal growth factor, 2 ng/mL human basic fibroblast growth factor, 5 μg/mL human insulin and 1% antibiotic-antimycotic solution).

The initial adhesion and proliferation of cells on the nanofiber sheets were measured by using a Cell Counting Kit-8 (CCK-8, Dojindo, Kumamoto, Japan) according to the manufacturer’s instructions. The cell viability was found to be directly proportional to the metabolic reaction products obtained in the CCK-8 assay [9,11]. Concisely, all cells were seeded on the pure PLGA and RGD-PLGA nanofiber sheets at a density of 1 × 10^4^ cells/mL, and each cell-seeded sheet was incubated with CCK-8 solution in the last 2 hours of the culture periods for the initial adhesion (6 hours) or proliferation (1, 3, 5 and 7 days) at 37 °C in the dark. Parallel sets of cells cultured on TCPs were regarded as the positive (+) controls. The absorbance was determined at 450 nm by using an ELISA reader (SpectraMax 340, Molecular Device Co., Sunnyvale, CA, USA).

### 3.4. Immunofluorescence Imaging of HASMCs on the RGD-PLGA Nanofiber Sheets

To investigate the adherent morphologies of HASMCs, the cells on the pure PLGA and RGD-PLGA nanofiber sheets for 3 days were fixed with 3.7% formaldehyde solution (Sigma-Aldrich Co., St Louis, MO, USA) for 10 minutes, and the cells were immersed in 0.1% Triton X-100 (Sigma-Aldrich Co.) for 5 minutes. Subsequently, the cells were blocked with 2% BSA solution in DPBS for 30 minutes and incubated with tetramethylrhodamine isothiocyanate (TRITC)-labelled phalloidin (200 units/mL in methanol, 1:40 in 1% BSA solution in DPBS, Molecular Probes, Eugene, OR, USA) for 20 minutes at RT. A 1 μM of DAPI (Sigma-Aldrich Co., St Louis, MO, USA) solution in DPBS was used for the nuclei counterstaining. To immunostain the decorated RGD-M13 phages, the HASMC-cultured sheets were stained with the primary and secondary antibodies, as described in Section 3.2. The stained HASMCs on sheets were imaged with a custom-built two-photon excitation fluorescence microscope [38,39].

## 4. Conclusions

The fabrication of artificial scaffolds for supporting cellular behaviors is one of the most attractive challenges in tissue engineering because of their potential for regenerating injured tissues. In the present study, the RGD-PLGA hybrid nanofiber sheets were fabricated by an electrospinning technique, and their characteristics were analyzed. It was revealed that the RGD-PLGA nanofiber sheets are composed of nanometer-scale diameters of hybrid fibers and have a three-dimensional porous architecture similar to natural ECM. The decoration of RGD peptides on the hybrid nanofiber sheets was successfully achieved by using RGD-M13 phages. In addition, their high surface area-to-volume ratio, hydrophilic surface property and suitable thermal behavior can provide a biocompatible microenvironment for cell growth. Moreover, we demonstrated that the cellular behaviors of four different types of cells on the RGD-PLGA nanofiber sheets were significantly promoted because of the combination of suitable physicochemical properties and the decorated RGD peptides. Therefore, our findings suggest that the RGD-PLGA hybrid nanofiber sheets are obviously effective for various cell types and are remarkably biocompatible and well designed to serve as cell-adhesive substrates. In summary, the RGD-PLGA hybrid nanofiber sheets are promising candidates that can be used as cell-adhesive substrates for tissue engineering scaffolds.
